# Automated Molecular Subtyping of Breast Carcinoma Using Deep Learning Techniques

**DOI:** 10.1109/JTEHM.2023.3241613

**Published:** 2023-02-06

**Authors:** S. Niyas, Ramya Bygari, Rachita Naik, Bhavishya Viswanath, Dhananjay Ugwekar, Tojo Mathew, J Kavya, Jyoti R Kini, Jeny Rajan

**Affiliations:** Department of Computer Science and EngineeringNational Institute of Technology Karnataka 119680 Surathkal 575025 India; Department of Computer Science and EngineeringThe National Institute of Engineering Mysuru 570008 India; Department of Pathology, Kasturba Medical College, MangaloreManipal Academy of Higher Education 76793 Manipal 576104 India

**Keywords:** Molecular subtyping, breast cancer, image segmentation, deep learning

## Abstract

Objective: Molecular subtyping is an important procedure for prognosis and targeted therapy of breast carcinoma, the most common type of malignancy affecting women. Immunohistochemistry (IHC) analysis is the widely accepted method for molecular subtyping. It involves the assessment of the four molecular biomarkers namely estrogen receptor (ER), progesterone receptor (PR), human epidermal growth factor receptor 2 (HER2), and antigen Ki67 using appropriate antibody reagents. Conventionally, these biomarkers are assessed manually by a pathologist, who finally combines individual results to identify the molecular subtype. Molecular subtyping necessitates the status of all the four biomarkers together, and to the best of our knowledge, no such automated method exists. This paper proposes a novel deep learning framework for automatic molecular subtyping of breast cancer from IHC images. Methods and procedures: A modified LadderNet architecture is proposed to segment the immunopositive elements from ER, PR, HER2, and Ki67 biomarker slides. This architecture uses long skip connections to pass encoder feature space from different semantic levels to the decoder layers, allowing concurrent learning with multi-scale features. The entire architecture is an ensemble of multiple fully convolutional neural networks, and learning pathways are chosen adaptively based on input data. The segmentation stage is followed by a post-processing stage to quantify the extent of immunopositive elements to predict the final status for each biomarker. Results: The performance of segmentation models for each IHC biomarker is evaluated qualitatively and quantitatively. Furthermore, the biomarker prediction results are also evaluated. The results obtained by our method are highly in concordance with manual assessment by pathologists. Clinical impact: Accurate automated molecular subtyping can speed up this pathology procedure, reduce pathologists’ workload and associated costs, and facilitate targeted treatment to obtain better outcomes.

## I. Introduction

Breast carcinoma is the most common cancer type worldwide and the leading cause of cancer-related deaths among women. As per the latest GLOBOCAN report [1], 2.3 million new cases of breast cancer and 6.84 lacs death cases were estimated globally in the year 2020. Breast cancer is a heterogeneous disease marked by the uncontrollable growth of malignant tumors that vary in their biological and clinical behavior. Accordingly, breast cancer is categorized by multiple bases as histological subtypes, molecular subtypes, functional subtypes [2] etc. Molecular subtyping of breast cancer helps in better prognostication [3] and targeted therapy [4] of the disease. Most studies classify breast cancer into four major molecular subtypes, namely Luminal A, Luminal B, Triple-negative/basal-like, and HER2-enriched [5]. This classification is based on the assessment of nuclear biomarkers such as estrogen receptor (ER), progesterone receptor (PR), antigen Ki67, and cell membrane marker Human Epidermal growth Receptor 2 (HER2). Hormonal receptors ER and PR present in the tumor cells accelerate the growth and division of cells in the presence of estrogen and progesterone hormones. In the IHC analysis, if ER/PR is above a certain threshold, the biomarker status is assigned as ER+/PR+. Ki67 is a protein found in growing/dividing cells but absent in the resting phase of cells. IHC analysis is used to measure the extent of this nuclear protein, which indicates the cell proliferation rate. HER2 is a protein that is responsible for the growth and repair of breast cells. Gene mutation causes overproduction of the HER2 protein (a state known as HER2 positive), leading to the rapid division of breast cells followed by mass formation.

The factors that influence the growth of different subtypes vary substantially. For the best outcome, each molecular subtype of cancer needs to be treated differently. The most common and cost-effective way of molecular subtyping is immunohistochemistry (IHC) analysis [6]. Other popular molecular testing approaches are fluorescent in situ hybridization (FISH) and gene expression profiling [7] that are costly and not widely available. IHC uses the principle of antibodies binding to specific antigens in biological tissues to detect the biomarkers’ presence. Based on its cost-effectiveness and universal availability, IHC analysis is considered as the gold standard for molecular subtyping and cancer prognostication [4]. Table 1 provides an account of the molecular subtypes, their prognostic characteristics, and treatment approaches of breast cancer.

**TABLE 1:** Molecular Subtypes, Diagnosis, and Prognosis of Breast Cancer (Compiled From [8])

Molecular Subtype	Ductal			
	Luminal		HER2 Enriched	Tripple Negative
	Luminal-A	Luminal-B		
Marker Response	ER+,PR+/-HER2-Low Ki67	ER-,PR+/-HER2+/-High Ki67	ER-,PR-HER2+High Ki67	ER-,PR-HER2-High Ki67
Prognosis	Good	Intermediate	Worse	Worse
Response to Chemo- therapy	Low	Intermediate	Higher	Higher
targeted Therapies	Hormonetherapy	Hormonetherapy	HER2Targetedtherapy	Underresearch

The conventional pathology procedure for molecular subtyping is predominantly a manual process. Here, the pathologists assess the aforementioned biomarkers in the chemically stained biopsy specimen of cancer patients by visual analysis through a microscope. The high-power field under observation is labeled as positive or negative for the three biomarkers (ER+/-, PR+/-, HER2+/-) based on visual analysis of the color response. For Ki67, a cell proliferation status as Low/Intermediate/High is assigned based on the number of Ki67 immunopositive cells. Once the individual assessments of all four biomarkers are completed, these outcomes are used to identify the molecular subtype, as shown in Table 1.

**FIGURE 1.**
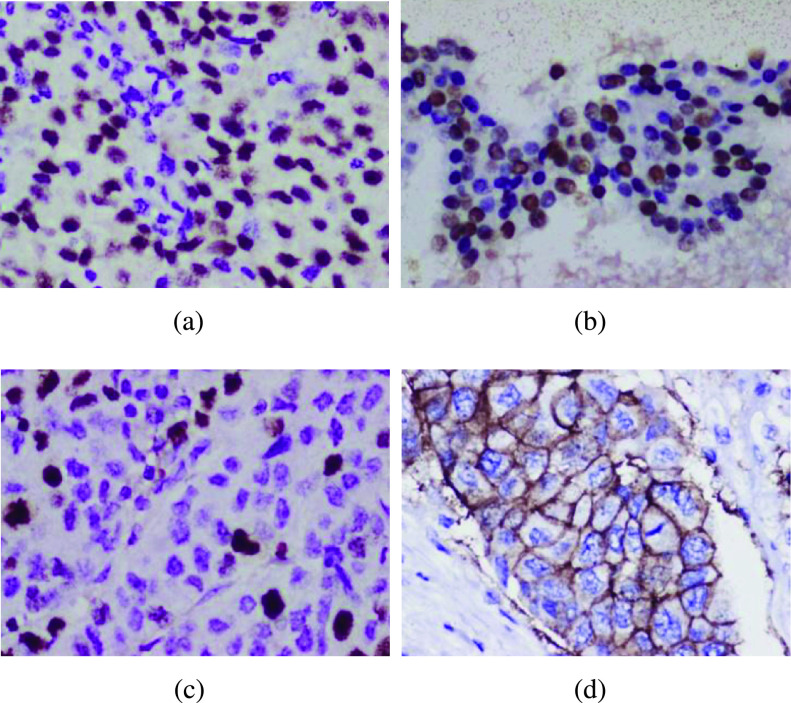
Representative IHC images of biomarkers used in molecular subtyping at 40X magnification (a) ER, (b) PR, (c) Ki67, and (d) HER2.

The manual procedure of molecular subtyping is tedious, error-prone, and has high inter-observer variability [9]. Digital pathology enables capturing and storage of biopsy slides in the form of digital images, and paves way for automating the process of molecular subtyping using image analysis algorithms. Fig. 1 demonstrates the samples of IHC slide images captured in this way. These images are then analyzed using computational algorithms to extract clinically relevant information.

Deep learning has been in the forefront of methodologies adopted in several works on medical image analysis recently [10], and they have given high performance, matching or even exceeding human-level accuracy. We used CNN based semantic segmentation models for the assessment of each of the histological biomarkers separately, based on the color intensity differences expressed by immunostaining. Using the standard guidelines and specifications [11], [12], [13], the final IHC biomarker status is assigned to samples of each biomarker. After this step, the molecular subtype is determined by combining the individual biomarker status according to the St Gallen International Expert Consensus on breast cancer [4].

The main contributions of this work can be summarized as follows.

1) A deep learning-based fully automated system is proposed for molecular subtyping of breast cancer using the IHC images of the biomarkers ER, PR, HER2, and Ki67.
2) An improved LadderNet architecture is proposed for the semantic segmentation of IHC images. The proposed architecture improves segmentation accuracy compared to the state-of-the-art architectures.

The proposed system to classify breast carcinomas based on immunohistochemical markers will reduce the manhours and report generating time. Conventionally, pathologists evaluate each immunomarker stained section/slide independently or as a consensus, collates all four markers’ data, and then comments on the final subtype. This is time-consuming and introduces interobserver variability unless reported as a consensus. The proposed system would speed up the process and spare the pathologist for higher-level interpretations. It would help overcome interobserver variability, promoting appropriate and timely patient care. The system would help pathologists and oncologists to plan hormone therapy/chemotherapy for individual patients, paving the way for precision medicine.

Rest of this paper is organized in the following manner. In Section II, a brief review of the related works from literature is carried out. Section III explains the methodology and the proposed architecture in detail. Experimental set up and obtained results are presented in Section IV along with related discussion. Section V concludes the paper with a discussion on the results and future work.

## II. Literature Review

Automated assessment of individual breast cancer biomarkers has attracted considerable research interest. However, to our knowledge, there is only one published work on automated molecular subtyping by analyzing all the four biomarker images [14]. In this method, a classification approach is adopted where nuclei-based patches are automatically extracted from the biomarker images and classified as +ve or –ve for the specific biomarker. Distribution of the nuclei-based patches is approximated as the actual distribution of nuclei in slide images. Since there are no other methods found for molecular subtyping, this literature review considers the methods reported for the individual biomarker assessment. Dhondalay et al. [15] used a 3-layer ANN to predict ER status of breast cancer from gene microarray data. Oscanoa et al. [16] used histogram thresholding to separate the background from the nuclei in an ER image, followed by segmentation and fuzzy c-means clustering. Overlapping nuclei are further detected using watershed segmentation. The method proposed by Mungle et al. [17] presented an automated Allred scoring model for screening ER images. The model used Markov random fields (MRF) with expectation maximization (EM) for cell segmentation and positive proportion scoring. The intensity score is computed with an ANN classifier, and both these scores are used to compute the final Allred score for ER. Progesterone receptor (PR) expression in breast tumors is similar to ER, and the same Allred system is used for determining PR status. Accordingly, Saha et al. [18] proposed a combined method for ER and PR status prediction based on deep learning (HscoreNet), which consists of three components, i.e., encoder, decoder, and scoring layer.

The biomarker Ki67 is an indicator of cell proliferation rate and hence tumor growth. Niazi et al. [19] used hotspot detection to classify Ki67 response images into immunopositive and immunonegative classes. This method considers hotspot detection as a clustering problem; hence several nuclei present together are counted as single nuclei and that affects the scoring accuracy. Saha et al. [20] identified the hotspots that form seed points in Ki67 images. The centroid of the seed point is used in patch selection such that only one nucleus exists in each patch. This is done using the expectation-maximization algorithm with a Gamma mixture model. CNN is further used to predict whether a patch is immunopositive or negative. Zhang et al. [21] used generative adversarial network (GAN) to generate more image samples for training. CNN-based image classification and object detection using a single shot multibox detector are used for Ki67 assessment. Narayanan et al. [22] used a VGG16 CNN model to extract sparse hyper column descriptors from selected convolutional layers to which the Ki67 image was fed as input. This pipeline can overcome the challenge of detecting weakly stained Ki67 negative nuclei. Dirican and Kilic [23] presented a retrospective investigation to cluster breast cancer prognostic factors based on the Ki67 score using machine learning algorithms. Lakshmi et al. [24] used a U-Net based segmentation to identify immunopositive and immunonegative nuclei, followed by connected component analysis for estimating the percentage of immunopositive cells and achieved a Dice score of 96%.

HER2 is a protein present in cell membranes that facilitates the proliferation of cells. Overexpression of HER2 is linked to tumor growth and forms an important factor in molecular subtyping. Tuominen et al. [25] proposed a color deconvolution-based method for HER2 status prediction and developed a free software application called ImmunoMembrane based on this method. Wdowiak et al. [26] presented another approach to discover small membrane sections defined by linear patterns of different shapes. This approach assumes that the complex shape of the membrane staining results from the small membrane sections. The method by Labellapansa et al. [27] assigns a score to HER2 samples as 1+ and 3+ based on the overexpression of HER2 protein. HER2 overexpression area percentage is calculated by dividing the HER2 positive area by the tumor area. Rodner et al. [28] used bilinear features introduced by Lin et al. [29] for HER2 scoring. AlexNet [30] pre-trained on the ImageNet dataset is used here to compute the bilinear features. A multi-class logistic regression is used to classify the four scoring classes. Saha and Chakraborty al. [31] proposed a deep learning model called Her2Net, consisting of two parts - convolution and deconvolution. The output of this model is a segmented mask consisting of cell membranes, nuclei, and background. The FC layer was used as a final layer or classification layer to assign scores to the HER2 sample. Mukundan [32] presented an approach for scoring HER2 samples based on key properties of four different types of features - texture features (through uniform local binary patterns), morphological features (to describe the connectivity of regions), the difference in stain intensity, and histogram statistics.

It is observed in our literature study that most of the existing methods for molecular biomarker status prediction of breast cancer address prediction of one or at most two of the four essential biomarkers of breast cancer molecular subtyping. Moreover, none of the existing methods target molecular subtyping as an outcome. In the proposed method, we address these research gaps in the current literature. We have collected samples of all the four biomarkers from multiple patients and implemented a comprehensive automated system for molecular subtyping of breast cancer, using deep learning-based image analysis. For the components ER and PR, our system follows the Allred scoring system [11], ASCO guidelines for HER2 [12] and for Ki67, recommendations from International Ki67 Breast Cancer Working Group [13]. Since the criteria for scoring and classification of each biomarker are different, we have developed separate customized deep learning models to analyze the image data, handle class imbalance, and extract the required elements from them. The proposed method is explained in the following section.

## III. Methodology

**FIGURE 2.**
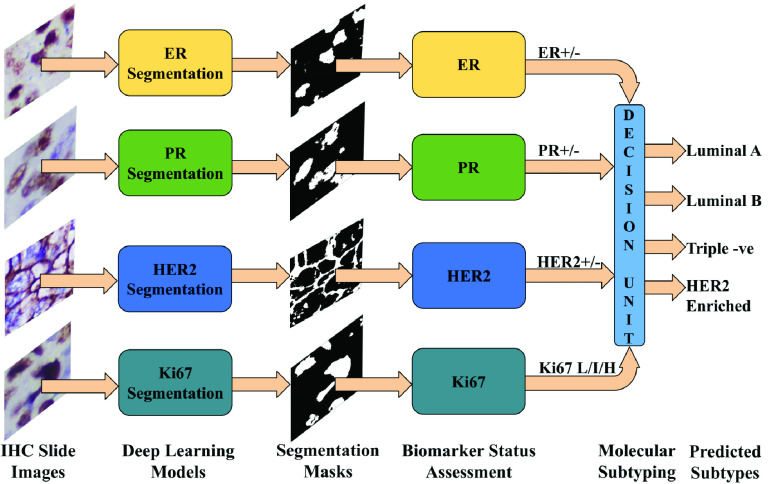
High-level breakdown of stages in automated molecular subtyping of breast cancer (L: Low, I: Intermediate, H: High).

Molecular subtyping of breast cancer requires the assessment of four important biomarkers’ presence in tumor tissues. The proposed method uses CNN based semantic segmentation to separate the relevant cellular elements such as nuclei and cell membranes that express the presence of these biomarkers. Image samples of biomarkers - ER, PR, Ki67, and HER2 are analyzed separately, and the response is assessed according to the clinical guidelines and the molecular subtype is determined as per the St. Gallen International Expert Consensus [4]. Fig. 2 presents a high-level operational overview of the proposed method. Since IHC images of the four biomarkers have different characteristics based on immuno-response, target objects etc., individually trained deep learning models are used for analyzing each biomarker type. During testing, the test image samples are passed as input to the trained deep learning models, and the corresponding multi-class segmentation masks are generated as output. These masks are then passed on to the respective biomarker status assessment modules to compute the score and predict the status of each biomarker. Finally, the decision unit combines the individual biomarker statuses to predict the molecular subtype.

### A. Data Acquisition and Pre-Processing

The proposed method uses digitized biopsy slides of biomarkers: ER, PR, HER2, and Ki67 images captured at 40X magnification. The dataset is collected from Kasturba Medical College, Mangalore, India, and it consists of 600 slide images from 15 breast cancer patients. There are 150 images per biomarker, and each image has a spatial resolution of $1920\times 1440$ pixels. Processing images at this resolution is computationally intensive, and hence, non-overlapping slices of size $480\times 480$ are created from each original image. These images are then resized to $240\times 240$ to provide a better trade-off between the segmentation accuracy and the computation overhead while training. Since there are 12 sliced image patches per IHC image, this process creates sufficient samples (1800 image samples from 150 images of each biomarker) to meet the training requirement of the deep learning models.

### B. Segmentation of Cell Elements from IHC Images

Color intensity differences in the IHC images express the presence or absence of targeted antigens in the corresponding biopsy specimen (Refer Fig. 1). In ER and PR slides, nuclei with a violet color indicate immunonegative cells, whereas solid dark-brown stained nuclei and granular dark-brown (or faint brown) nuclei correspond to strong and weak positive cells, respectively. So, A four-class segmentation approach (background, weak positive, strong positive, and immunonegative) is used ER and PR stains for segmenting cells. In Ki67 images, the immunopositive cells appear brownish-red, whereas the immunonegative cells appear violet, so a three-class segmentation approach (background, immunonegative, and immunopositive) is followed for Ki67 samples. In HER2 images, the nuclei appear blue-violet while the cell membrane appears brown. Hence, a three-class segmentation approach (background, nuclei, and membrane) has been used in the case of HER2 images.

The final biomarker scoring is highly influenced by the accuracy of estimating the immunopositive and immunonegative cell elements from the IHC images of the biomarkers. Though the number of target classes is different for the biomarkers, the IHC slide images share some common characteristics such as the shades in biomarker color response and the background color. Hence, the proposed semantic segmentation model is designed to segment immunopositive elements from all four IHC image types. The model uses an improved LadderNet architecture, consisting of two encoder-decoder U-Net modules connected serially with customized skip connections and convolution blocks.

#### 1) Network Architecture:

The proposed segmentation model is inspired from the LadderNet architecture [33] that uses a series of connected U-Net [34] modules. We have experimented with several popular standard deep learning architectures such as U-Net, ResNet, DenseNet, U-Net++, and LadderNet and their derivatives to select an appropriate model for the IHC segmentation. Among these models, LadderNet has given the best segmentation performance. We further optimized the performance by incorporating different design choices into the baseline LadderNet architecture. The concatenated skip connections in the U-Net model across the encoder-decoder branches help to pass information across the layers. The number of learnable paths and information flow across a U-Net model is limited due to fewer skip connections. In addition, the semantic gap between the feature space at an encoder and decoder level is higher (especially at lower depths) and can lead to adverse effects in the learning process. Basic LadderNet addresses many of these issues by providing multiple pairs of encoder-decoder branches. However, decoder modules in each depth use feature space from the corresponding encoder depth and hence limit the information flow from the encoder and decoder paths.

**FIGURE 3.**
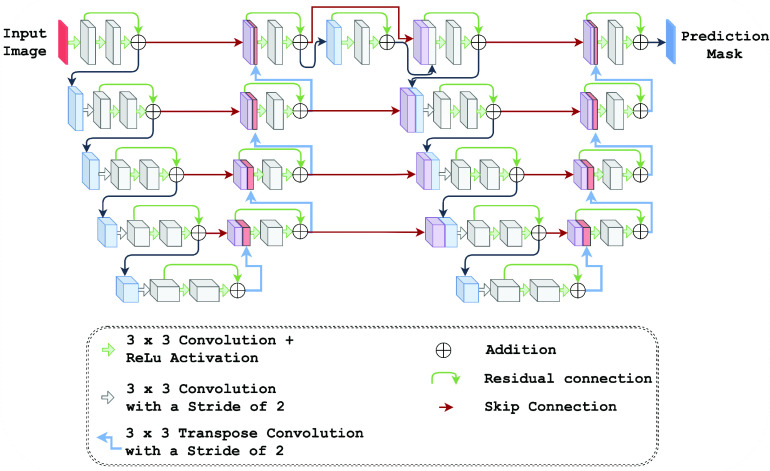
The proposed CNN architecture for IHC image segmentation.

The proposed model aims at providing more learnable paths to the basic LadderNet architecture. The architecture of the proposed segmentation model is represented in Fig. 3. This network uses concatenated feature space from different semantic levels that allows the model to learn from different scales of the image data. The expansive path of the proposed model at level $l$ uses a concatenated feature space from:

1) A downsampled output from $(l-1)^{th}$ level encoder.
2) Encoder output from the same level $l$.
3) An upsampled output from $(l+1)^{th}$ level encoder.
4) An upsampled and deconvoluted output from $(l+1)^{th}$ level decoder.

The additional skip connections in the proposed architecture also create more data flow paths. Hence, the overall architecture acts as an ensemble of multiple fully convolutional neural networks and the learning paths are adaptively selected based on the nature of input image samples. The multi-scale feature concatenation and the adaptive selection of learning paths provide an optimal segmentation performance over different types of images. These benefits of the proposed architecture help to use the same architecture to segment the four types of biomarker images. The architecture also uses residual convolution blocks for feature extraction and strided convolution for downsampling feature space in the encoding path.

Class imbalance is a major challenge faced in the current IHC image analysis. The regions of pathological relevance typically occupy only a small image area, leading to instability in the learning pattern. The dataset shows a skewed ratio of pixels belonging to different classes. Among the target classes, the pixel distribution is quite unbalanced and, in the order, background $\gg $ strong $\gg $ intermediate $\gg $ weak. For instance, the average pixel ratio of background, strong, intermediate, and weak classes in the ER images are 141:14:6:1. To reduce the adverse effect of class imbalance, the proposed method uses a customised loss function (Eqn. 1) which is a combination of Categorical Cross-Entropy ( ${CCE}$) and Focal Tversky Losses: ${FTL}_{1}$ that penalizes false positives and ${FTL}_{2}$ that penalizes false negatives.

\begin{align*} F_{loss} &= (0.2 \times CCE) + 0.4 \times (FTL_{1} + FTL_{2}) \tag{1}\\ FTL_{1} &= {(1-{TI}_{1})}^{(\gamma)}; FTL_{2} = {(1-{TI}_{2})}^{(\gamma)} \tag{2}\\ {TI}_{1} &= \frac {TP}{TP + \left ({0.75 \times FP }\right) + \left ({0.25 \times FN}\right)} \tag{3}\\ {TI}_{2} &= \frac {TP}{TP + \left ({0.25 \times FP }\right) + \left ({0.75 \times FN}\right)} \tag{4}\end{align*}

where $TP$, $FP$, and $FN$ stand for true positives, false positives, and false negatives, respectively and ${TI}_{1}$ and ${TI}_{2}$ represent the Tversky similarity indices with different weights for $FP$ and $FN$.

Focal Tversky Loss (FTL) allows better control over the learning performance using the parameter $\gamma $. In IHC analysis, estimating the different classes of cell elements is more critical than pixel-level segmentation. FTL becomes useful in such cases with $0< \gamma < 1$. This results in a higher loss gradient for samples with $TI > 0.5$, which leads to better generalized learning. Hence, the combination of CCE and two FTL components helps adequate learning of the image characteristics.

In the proposed architecture, both the left and right encoder-decoder modules use 16 filters each at level 0, and the number of filters in each subsequent level is increased by a factor of two. All convolution and deconvolution layers (except the final layer) use ReLU activation followed by batch normalization, and SoftMax activation is used in the final classification layer. The input layer that accepts images of size $240 \times 240 \times 3$ is common to all four biomarker models. The output layer is defined based on the number of segmentation classes in each IHC biomarker. Hence for training and testing, input images and corresponding ground truth masks are sliced into non-overlapping patches of size $240 \times 240$. After patch-wise segmentation, the prediction masks of all the patches from an IHC image are stitched together to create the final prediction mask corresponding to the image.

### C. Post-Processing

The segmentation stage extracts target objects such as different classes of nuclei and cell membrane from IHC images. The segmentation output may contain noise elements and overlapped cells. Hence, a post-processing stage is introduced to reduce the misclassified pixels from the segmentation output and separate the target objects in each image. We applied the watershed segmentation method [35] followed by morphological operations (opening and closing) to split overlapping nuclei and remove tiny pixel patches from the segmentation output.

### D. Biomarker Scoring, and Decision Making

The biomarker scoring for each IHC image is conducted separately for the molecular subtype prediction. In the case of ER and PR images, the counts of positive nuclei (strong, intermediate, weak) and negative nuclei are obtained using connected component analysis of the segmented mask. The Allred Scoring system is used to determine the ER/PR status, where each sample is assigned with a Proportion Score (PS) and an Intensity Score (IS) [11], [36]. Tables II and III show the criteria for assigning proportion and intensity scores to a sample. The total score (TS) is then computed as the sum of PS and IS. When TS < 3, the sample status is negative and vice versa. Following this criterion, each ER and PR sample is identified as positive or negative. Multiple image slides from a patient are examined and hence use a majority voting scheme to obtain the final patient-wise biomarker status of both ER and PR biomarkers.

**TABLE 2:** ER/PR Intensity Score Calculation

ProportionScore	ER/PR+ve cells
0	0%
1	< 1%
2	1–10%
3	11–33%
4	34–66%
5	67–100%

**TABLE 3:** ER/PR Intensity Score Calculation

IntensityScore	ER/PRIntensity
0	Negative
1	Weak
2	Intermediate
3	Strong

In the case of Ki67, the counts of immunopositive and immunonegative nuclei are obtained using connected component analysis of the Ki67 prediction mask. Then the Ki67 proliferation index (PI) is calculated using Eqn. 5.

\begin{equation*} PI=(N_{p} \times 100)/ (N_{p} +N_{n}) \tag{5}\end{equation*}

where $N_{p}$ and $N_{n}$ are the number of immunopositive and immunonegative nuclei present in each slide. Based on the PI value, each image sample is assigned with a Ki67 proliferation status as *low* (PI < 14%), *intermediate* (14% $\leq $ PI $\leq21$%), or *high* (PI $>$ 21%). The patient’s Ki67 proliferation status is also obtained by the majority voting scheme of the image-wise results.

HER2 scoring is based on two factors - *intensity* and *completeness* of the cell membrane, as per the American Society of Clinical Oncology (ASCO) [12]. *Intensity* is obtained using a shallow CNN classifier which takes the HER2 image as input and classifies the membrane as faint or intense. The *completeness* of cell membranes is determined by computing the percentage of membrane over a specific radius outside each nucleus. The final HER2 scoring is made based on the *intensity* and *completeness* of the cell membrane as shown in Table 4. HER2 status for a patient is then assigned based on the majority voting scheme of the image-wise results.

**TABLE 4:** HER2 Scoring Based on Cell Membrane Features

% Cells	Completeness	Intensity	HER2Score	HER2Status
< = 10%	None/Incomplete	Faint	0	Negative
$>$10%	Incomplete	Faint	1+	Negative
$>$10%	Moderatelycomplete	Intense	2+	Equivocal
$>$10%	Complete	Intense	3+	Positive

After computing the patient-level biomarker statuses for ER, PR, HER2, and Ki67, they are combined to determine the molecular subtype, based on the medical guidelines for treatment planning as shown in Table 1.

## IV. Experimental Results & Discussion

The experimental setup, training methodology, ablation study over different design choices in the proposed architecture, quantitative and qualitative results, and the performance comparison of the proposed model with state-of-the-art IHC biomarker analysis methods are included in this section.

### A. Experimental Setup

All experiments are conducted using the Google Colab environment and NVIDIA®DGX-® machine loaded with Canonical Ubuntu OS, Dual 20-Core Intel®Xeon E5-2698 v4 CPU @2.2 GHz, 512 GB of RAM, and 8X NVIDIA®Tesla®V100 GPU with 32GB dedicated memory.

### B. Training Methodology

The proposed deep CNN model of depth 4 with an input size of $240 \times 240 \times 3$ is used for segmentation of different classes of nuclei and cell membranes. The hyperparameters, such as the number of filters, depth, dropout level, loss parameters etc., are empirically selected based on the performance in multiple experiments carried out. For each biomarker, experiments are separately conducted using 150 slide images per biomarker collected from 15 patients. Since the patient samples are low, leave-one-out cross-validation (LOOCV) is used to guarantee an unbiased performance evaluation. In each fold, all images from one patient are used for testing and the remaining 14 patients’ images as the training data. This was repeated 15 times, and average performance across the 15 folds is taken as the final result. The same procedure is repeated for all four biomarkers by training from scratch to generate the trained models. Though the training set consists of only 140 images in each IHC type, after slicing them into patches of size $240 \times 240$, there are 1680 training image samples to make a sufficient training set. Hence in each fold, 140 images from 14 patients are used for training, and ten images from the remaining patient are used for testing.

Best performance is observed using a batch size of four and dropout (with a rate of 0.1) in the decoding layers. $L2$ regularization has been used to avoid overfitting, and Adam optimizer is used with a learning rate = 0.001. The *He normal initializer* is used for initializing kernel weights in all segmentation models, and each model is trained from scratch for 50 epochs.

### C. Results & Discussion

**FIGURE 4.**
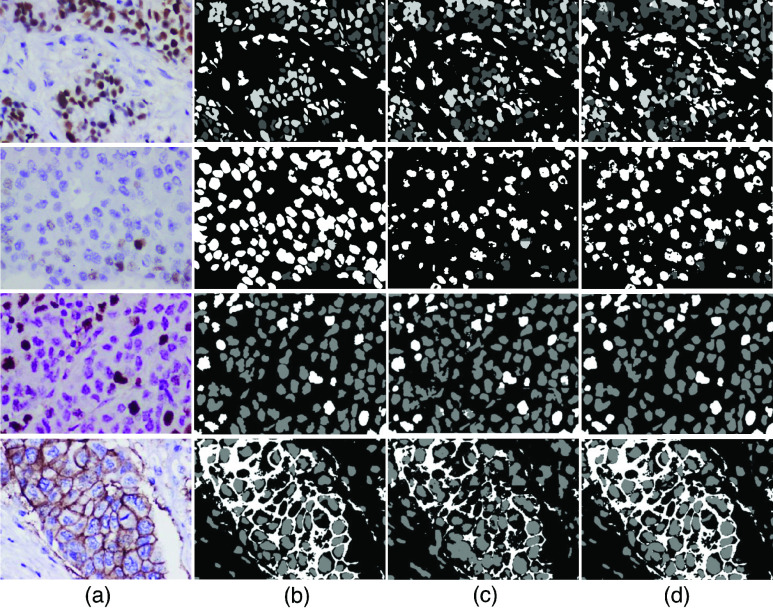
Qualitative analysis of the immunopositive cell segmentation: (a) Biomarker image samples of ER, PR, Ki67 & HER2 (from top), (b) Ground truth, (c) Predicted outcomes from the state-of-the-art (HScoreNet [18] for ER & PR, and [24,34] for Ki67 and HER2, respectively), and (d) Predicted outcomes from the proposed method.

The performance of segmentation models for each IHC biomarker is evaluated qualitatively and quantitatively. Qualitative analysis of ER, PR, and Ki67 segmentation involves verifying whether all the immunopositive nuclei are segmented and classified into the correct classes specified in the ground truth. In HER2, the nuclei and cell membrane need to be accurately segmented without obscuring each other. To our knowledge, no automated methods for molecular subtyping based on all four IHC images have been found in the literature, and thus we compared our results to state-of-the-art segmentation models in each biomarker category. For ER and PR, we compared the result of our improved LadderNet with the state-of-the-art HScoreNet [18]. For Ki67 and HER2, the segmentation results are compared with U-Net based approaches [24], [34] in their respective domains. Fig. 4 shows the segmentation performance of the proposed model and the state-of-the-art methods. The proposed model results are closer to the ground truth, and the false predictions across the subclasses are relatively lesser. Pixel-wise analysis is one of the popular methods for evaluating semantic segmentation. The pixel-wise cross-validation results for each IHC biomarker are reported in Table 5.

**TABLE 5:** Pixel-Wise Evaluation of the Segmentation Results

IHCImageType	Method	Sub-classes	Evaluation Metrics		
			Preci-sion	Recall	DiceScore
ER	Proposed Method	Strong positive	0.66	0.67	0.66
		Weak positive	0.45	0.56	0.50
		Immunonegative	0.59	0.69	0.63
	Hscore Net [18]	Strong positive	0.64	0.66	0.65
		Weak positive	0.47	0.43	0.45
		Immunonegative	0.61	0.63	0.62
PR	Proposed Method	Strong positive	0.59	0.76	0.67
		Weak positive	0.52	0.61	0.56
		Immunonegative	0.67	0.77	0.72
	Hscore Net [18]	Strong positive	0.59	0.63	0.61
		Weak positive	0.55	0.56	0.56
		Immunonegative	0.63	0.66	0.64
Ki67	Proposed Method	Immunopositive	0.86	0.80	0.83
		Immunonegative	0.82	0.87	0.84
	Lakshmi et al. [24]	Immunopositive	0.76	0.71	0.73
		Immunonegative	0.81	0.85	0.83
HER2	Proposed Method	Nuclei	0.74	0.70	0.72
		Membrane	0.95	0.90	0.93
	Ronneberger et al. [34]	Nuclei	0.66	0.83	0.73
		Membrane	0.96	0.86	0.90

Molecular subtyping depends on detecting responses of cell elements like nuclei and cell membrane to IHC reagents and estimating the number of nuclei belonging to different classes is highly significant. Hence, an object-wise evaluation is also performed to check the ability of the model to predict the cell elements correctly. The object-wise results and the biomarker status prediction performance are shown in Table 6. The proposed model shows a fair trade-off between Precision and Recall while detecting the most significant cell elements in all biomarkers. Correctly estimating the cell elements leads to a reliable prediction of biomarker status for each slide and eventually leads to a molecular subtyping accuracy of 93.3% across all patients.

**TABLE 6:** Object-Wise Evaluation Results

IHCImageType	Object-wise Analysis				BiomarkerResponseAccuracy
	Sub-classes	Preci-sion	Recall	DiceScore	
ER	Strong positive	0.79	0.81	0.80	93.3%
	Weak positive	0.69	0.78	0.73	
	Immunonegative	0.74	0.83	0.78	
PR	Strong positive	0.86	0.92	0.89	93.3%
	Weak positive	0.79	0.88	0.83	
	Immunonegative	0.80	0.94	0.86	
Ki67	Immunopositive	0.86	0.91	0.88	100%
	Immunonegative	0.91	0.92	0.92	
HER2	Nuclei	0.90	0.88	0.89	85.7%

The encouraging results obtained for the proposed system point to the possibility of its further development and fine-tuning towards routine clinical application. Implementation of the proposed system in routine clinical practice can be done via integration with software systems currently in use with microscopes for slide image acquisition and analysis, or as an independent application. This system can run on the same computer system associated with the microscope in small clinics, or it can be deployed in an intranet or cloud environment to cater to the needs of large clinics and distributed chain of clinics. Amendments are required in the existing pathology procedures to enable the use of such systems in clinical practice. Regulatory agencies like the USA’s Food and Drug Agency (FDA) [37] and the UK’s Medicines and Healthcare Products Regulatory Agency (MHRA) [38] are already in the forefront of driving such regulatory changes and clinical process definitions. Generally, AI based models have the characteristics of incremental and continuous improvement since such models continue to learn on exposure to more and more labeled samples. Hence, in situ evaluation and fine tuning of the model can go hand-in-hand until the desired performance is achieved for the system before it is actually deployed in clinical practice.

## V. Conclusion

Automatic molecular subtyping of breast cancer leads to better prognostication and targeted therapy by avoiding several issues with the manual procedure. This article proposes a deep learning-based system for molecular subtyping of breast cancer using IHC biomarker images. A CNN architecture is proposed to segment various cell elements from digitized IHC images of the breast tissues that express the presence of molecular biomarkers ER, PR, Ki67, and HER2. After segmentation, post-processing based on connected component analysis is performed to count the cell elements that belong to different classes, followed by the biomarker score computation. By individually analyzing all four biomarkers from the same patient, the proposed method emulates the routine procedure followed by a pathologist while eliminating the inherent problems in manual analysis. The segmentation performance of the proposed model is compared with state-of-the-art approaches and demonstrates the improvement in the segmentation performance. Being one of the pioneering attempts to automate molecular subtyping using IHC image analysis, we see tremendous potential for future enhancements in this task. With more data, it is possible to improve the segmentation performance further to develop automated assistive technologies for clinical trials.
